# What’s Next for Smart Implants in Health Care?

**DOI:** 10.2196/87975

**Published:** 2025-11-26

**Authors:** Mark Crawford

**Keywords:** microelectronic implants, monitoring, physiologic, technology, biomedical, biomedical engineering, telemetry, artificial intelligence, delivery of health care

**Key Takeaways**

- The uses of smart implants—medical devices with increasingly advanced embedded sensors and data analytics capabilities—are expanding.
- These devices have the potential to transform patient care and improve health outcomes by enabling earlier detection of complications; more proactive, data-driven clinical decisions; and improved operational efficiencies.

In the early 1900s, orthopedic surgeons began exploring the possibility of replacing damaged joints with implants made from artificial materials [1]. The first successful hip implant surgeries occurred in the early 1960s [2]. Implants have since evolved from passive tissue replacement to include microelectronic sensors and data analytic capabilities allowing continuous transmission and analysis of performance and physiological data.

From orthopedics to dentistry and beyond, these “smart” implants can play an increasingly active role in patients’ treatment, improving patient care and reducing operational costs [3].

## Smart Implant Uses

Smart implants have many current and potential applications. In a recent perspective article [4], Jincai Huang and a multidisciplinary team at Tsinghua University, Beijing, China, note that the incorporation of sensor technology “offers a new dimension for quantitative biomechanical assessment, enhancing clinical outcomes and reducing complications.”

For example, postsurgical infections and device failures affect up to 10% [5] of patients, and the removal of malfunctioning or infected joint implants is costly and painful. Smart implants equipped with embedded sensors can identify small abnormal changes in implant performance earlier, allowing surgeons to make repairs and restore functionality, often avoiding the trauma associated with a second surgery.

Smart implant applications have also expanded with the addition of artificial intelligence–driven data analytics and algorithms, which can help identify subtle trends and provide insights to support clinical decision-making. Through continuous monitoring and analysis, research [3,6] suggests that smart implants can enhance:

- Treatment delivery and personalization by allowing, for example, medication release triggered by changes in real-time physiological data; personalized rehabilitation programs based on patient movements and daily activity levels; and better treatment delivery, adherence, and management of chronic conditions.
- Access to care and operational efficiency with, for example, wireless-enabled remote monitoring, helping to reduce hospital visits and costs and improve access to care for those in remote communities or with limited mobility.
- Future implant design by providing insights into the mechanical and biochemical performance of implants over time, leading to improved materials, electronics, and designs.

## Barriers to Development

Several challenges, especially regarding patient safety and data security, must be resolved to facilitate the development and widespread implementation of smart implants.

One major concern is the infrastructure investment required to integrate smart implants into existing health care systems. Ensuring interoperability and upgrading electronic health records to handle the type, volume, and frequency of data generated can be costly and complex [3,7]. It requires, for example, supporting and standardizing new data formats and developing compatible interfaces to store and synchronize data.

Another key issue is keeping up with the technology and oversight required to protect patient privacy and data security. “Regulatory frameworks must evolve to address the unique complexities of smart implants,” noted Omid Panahi, Professor of Dentistry at Centro Escolar University, Manila, Philippines, in a recent review [3]. “Ethical considerations, such as data ownership, algorithmic bias, and liability, require careful deliberation and the establishment of clear guidelines.”

Other long-standing challenges noted in Panahi’s review [3] include ensuring long-term biocompatibility to minimize the risk of adverse reactions and implant rejection or failure. Another key consideration is the development of sensors that are sufficiently small, adequately powered, and robust against corrosion, stress, and biofouling (accumulation of biological material on the implant).

## Ongoing Innovation

Despite these challenges, applications for smart implants continue to expand. “Orthopedics is most popular because load and alignment are critical in knees, hips, spine, and trauma plates,” notes Dr Amir Alavi, Assistant Professor of Civil and Environmental Engineering and Director of the Intelligent Structural Monitoring and Response Testing (iSMART) Lab at the University of Pittsburgh. “Cardiac and gastrointestinal are next for monitoring pressure and motility. Dentistry is exploring occlusal force and healing. Smart implant use cases for pain and neuromodulation also exist.”

**Figure:**
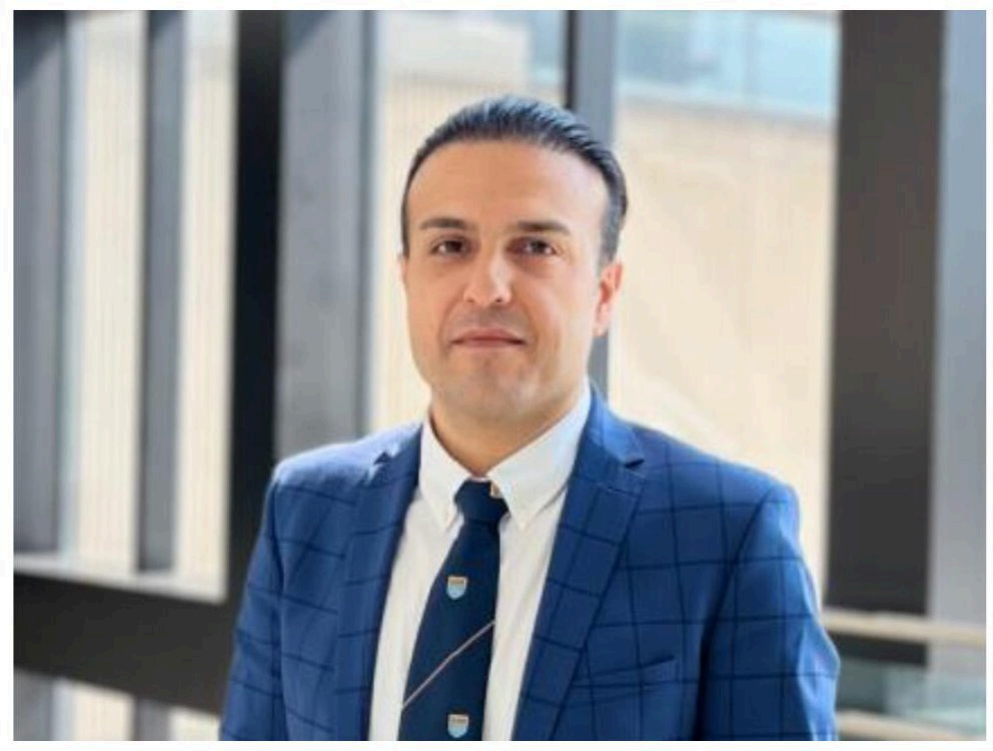
Dr Amir Alavi

Dr Alavi has developed a new class of metamaterials [8] that act as their own sensors, recording and relaying important information about the pressure and stresses on their structures.

“It is very challenging to integrate bulky circuits or power sources into the small area of implants,” says Dr Alavi. “Our new material is designed such that, under pressure, contact-electrification occurs between their conductive and dielectric microlayers, creating an electric charge that relays information about the condition of the material matrix of the implant.”

Dr Alavi feels this is a breakthrough.

“It shows how a metamaterial implant can function as a mini-router in vivo, like a passive structure capable of transmitting signals from inside to outside the body without any internal power source, batteries, or active communication hardware,” he says.

Another potential application is biodegradable smart implants. As noted in Huang and team’s article [4], “Leaving orthopedic fixators in the body long-term can lead to stress shielding and stress concentration, potentially causing localized osteoporosis or complex fractures around the implant. However, surgical removal of the fixators carries a high risk for complications, including infection, nerve injury, and re-fracture.” To solve this dilemma, researchers have proposed biodegradable material–based orthopedic implants [9], which can eliminate the need for secondary surgery and avoid complications caused by retained implants.

## Transforming Patient Care

Ultimately, smart implants have the potential to transform patient care—improving health outcomes, reducing hospital stays, boosting treatment adherence, personalizing treatment strategies, and helping manage complex or chronic conditions [3,4,6,10].

“Reimbursement policies are also starting to favor remote patient monitoring, which further supports acceptance of smart implants,” says Dr Alavi.

Through on-site fabrication, improved workflows, and other operational efficiencies, smart implants can relieve some of the major cost pressures on health care systems today. Continued research momentum, investment in infrastructure, and advances in materials will be essential to fully realizing their promise.
